# Interaction Between Cecal Metabolites and Liver Lipid Metabolism Pathways During Induced Molting in Laying Hens

**DOI:** 10.3389/fphys.2022.862721

**Published:** 2022-05-20

**Authors:** Jun Zhang, Xiaoqing Geng, Yihui Zhang, Xinlong Zhao, Pengwei Zhang, Guirong Sun, Wenting Li, Donghua Li, Ruili Han, Guoxi Li, Yadong Tian, Xiaojun Liu, Xiangtao Kang, Ruirui Jiang

**Affiliations:** ^1^ College of Animal Science and Technology, Henan Agricultural University, Zhengzhou, China; ^2^ Henan Innovative Engineering Research Center of Poultry Germplasm Resource, Henan Agricultural University, Zhengzhou, China

**Keywords:** induced molting, lipid metabolism, liver, cecum, hens

## Abstract

Moult is a normal physiological phenomenon in poultry. Induced molting (IM) is the most widely used and economical molting technique. By inducing moult, the laying hens can grow new feathers during the next laying cycle and improve laying performance. However, the lack of energy supply has a huge impact on both the liver and intestines and acts on the intestines and liver through the “gut-liver axis”. More importantly, lipid metabolism in the liver is closely related to the laying performance of laying hens. Therefore, in this study, cecal metabolites and liver transcriptome data during IM of laying hens at the late stage of laying (stop feeding method) were analyzed together to reveal the regulatory mechanism of “gut-liver axis” affecting the laying performance of laying hens from the perspective of lipid metabolism. Transcriptome analysis revealed that 4,796 genes were obtained, among which 2,784 genes had significant differences (*p* < 0.05). Forty-nine genes were associated with lipid metabolism, and five core genes (*AGPAT2*, *SGPL1*, *SPTLC1*, *PISD*, and *CYP51A1*) were identified by WGCNA. Most of these differential genes are enriched in steroid biosynthesis, cholesterol metabolism, drug metabolism—cytochrome P450, synthesis and degradation of ketone bodies, PPAR signaling pathway, and bile secretion. A total of 96 differential metabolites were obtained by correlating them with metabolome data. Induced moult affects laying performance by regulating genes related to lipid metabolism, and the cecal metabolites associated with these genes are likely to regulate the expression of these genes through the “enterohepatic circulation”. This experiment enriched the theoretical basis of induced moult and provided the basis for prolonging the feeding cycle of laying hens.

## 1 Introduction

Molting is a natural physiological phenomenon of birds in response to seasonal changes (Abg, 2008). During molting, the ovaries deteriorate and estrogen production decreases, resulting in reduced laying capacity and egg production (Brake, 1993). Natural molting generally needs 4 months and lasts a long time without uniform production time, which seriously affects the economic benefits of operators (Belland, 2003). However, Induced molting (IM) can shorten the molting time, synchronize egg production, save breeding costs, and improve egg production performance in the next cycle (Breeding et al., 1992; Alodan and Mashaly, 1999; Berry, 2003; Sandhu et al., 2007).

IM refers to the intense and sudden stress response caused by humans to chickens, resulting in nutritional disorders, metabolic disorders, endocrine disorders, and promoting the rapid molting of chickens to resume egg production (Zhang, 2021). There are many ways to force molting, but fasting is the most popular because it is simple and less expensive (Onbaşılar and Erol, 2007).

Studies have shown that when nutrients are deprived, the body’s glucose is initially provided from the stores of glycogen, but glycogen is quickly depleted (Furchtgott et al., 2009). If fasting continues, fatty acids become the main source of energy. Lipids break down the produced and released non-esterified fatty acids (NEFAs) and glycerol. NEFAs are oxidized to ketone bodies (ketogenesis) in the liver mitochondria through fatty acid D. Glucose and ketone bodies produced by the liver are the main metabolites of extrahepatic tissues and organs during starvation and exercise (Li et al., 2001; Arai et al., 2003).

During fasting, the gut, as an important place for digestion and nutrient absorption (Dou et al., 2002), loses the supply of nutrients and energy, and then the morphological and physiological characteristics of the gut and the homeostasis of intestinal microbes are greatly changed (Michalsen et al., 2005; Kohl et al., 2014; Gebert et al., 2020), which directly or indirectly affects the health and production performance of animals (Ferraris and Carey, 2000). There are a large number and a wide variety of microbial communities in the gastrointestinal tract of poultry, and the cecum is an important place for the survival and activity of microorganisms in the digestive tract of poultry (Zhen, 2019). The cecum is in an anerobic environment for a prolonged period, making it a fermenter for some anerobic bacteria, so it has the function of preventing the colonization of pathogenic bacteria and promoting intestinal health (Gérard, 2008).

Unlike mammals, lipid metabolism in poultry takes place mainly in the liver (Butler, 1975; Szabo et al., 2005). Although poultry adipose tissue can also esterify a small amount of fatty acids into triglycerides, it is not the main tissue of poultry triglyceride (TG) production (Leveille et al., 1975; Brady et al., 1976; Bedu et al., 2002). In order to meet the high demand for TG and cholesterol during laying, the liver of laying hens is particularly active in fat synthesis (Klasing, 1998) because yolk formation requires the transport of large amounts of hepatic lipoproteins to the developing oocytes of laying hens, whereas the ovaries of laying hens do not synthesize lipids. Fasting reduces fat production in the liver, cutting off the oocyte’s fat source.

With the development of multi-omics, transcriptome has been widely used in genetic breeding and nutritional regulation of chicken (Li et al., 2013; Li et al., 2018; Wang, 2019; As, 2021; Luo et al., 2021), however, few researchers have focused on lipid metabolism during IM. On the one hand, numerous studies have shown that intermittent fasting benefits human and animal health through lipid metabolism, significantly improving fatty liver and non-alcoholic fatty liver disease (David, 2014; Patterson et al., 2015). The gut and liver, on the other hand, are closely related in embryonic origin and anatomy and interact through the “gut-liver axis” (Compare et al., 2012; Paolella, 2014; Hussain et al., 2020). Therefore, based on the existing studies, this study analyzed the liver transcriptome and cecal metabolome of laying hens and revealed the interaction between the changes of cecal metabolites induced by hunger and liver lipid metabolism, and the effect of intestinal microbes on the laying performance of laying hens during IM. More importantly, it provides a theoretical reference for the study of IM.

## 2 Materials and Methods

### 2.1 Experimental Animals and Sampling

Ninety lady chickens at the late stage of laying (500 days of age) were selected and divided into nine replicates with 10 chickens in each replicate. According to the compulsory molting procedure, using the timeline as a control, there are six key time points in this experiment (Table 1, namely, F0 (on the day before the first day of feed breaking); F3 (on the third day of feed breaking); F16 (on the 16th day of feed breaking); R6 (on the sixth day of feed resuming); R16 (on the 16th day of feed resuming); and R32 (on the 32nd day of feed resuming). The samples (liver tissue samples and cecal contents) were collected at each treatment period, and sequencing of the liver transcriptome and cecal contents metabolome was commissioned by Gene Denovo Biotechnology Co., Ltd., Guangzhou.

**TABLE 1:** IM program induced by starvation.

Test period	Treatment			
	Feed	Water	Light	Processing time for each stage
F0	Normal feed	√	16 h	On the day before the test
F3	No feed	×	8 h	On the third day of fasting
F16	No feed	√	10 h	On the 16th day of fasting
R6	Gradually resuming feeding	√	10 h + 0.5 h per day	On the sixth day of recovery
R16		√		On the 16th day of recovery
R32	Normal feed	√	16 h	On the 30th day of recovery

### 2.2 Transcriptome Analysis

#### 2.2.1 RNA Extraction, cDNA Library Construction, and Sequencing

Total RNA was extracted using a TRIzol reagent kit (Invitrogen, Carlsbad, CA, United States) according to the manufacturer’s protocol. RNA quality was assessed on an Agilent 2,100 Bioanalyzer (Agilent Technologies, Palo Alto, CA, United States) and checked using RNase-free agarose gel electrophoresis. After total RNA was extracted, eukaryotic mRNA was enriched by oligo (dT) beads, while prokaryotic mRNA was enriched by removing rRNA by the Ribo-ZeroTM Magnetic Kit (Epicentre, Madison, WI, United States). Then, the enriched mRNA was fragmented into short fragments using fragmentation buffer and reverse-transcripted onto cDNA with random primers. Second-strand cDNA was synthesized by DNA polymerase I, RNase H, dNTP, and buffer. Then, the cDNA fragments were purified with a QiaQuick PCR extraction kit (Qiagen, Venlo, Netherlands), end-repaired, poly(A) added, and ligated to Illumina sequencing adapters. The ligation products were size-selected by agarose gel electrophoresis, PCR-amplified, and sequenced using Illumina HiSeq2500 by Gene Denovo Biotechnology Co. (Guangzhou, China).

#### 2.2.2 Filtering of Clean Reads, Alignment With the Reference Genome, and DEG Analysis

Reads obtained from the sequencer contain adapters or raw reads of low-quality base, which will affect subsequent assembly and analysis. Therefore, for clean reads of high quality, reads that contain the adapter should be removed; reads (N) containing more than 10% unknown nucleotides were removed; low-quality reads containing more than 50% of low-quality (Q ≤ 20) bases were removed (Chen et al., 2018). The short fragment comparison tool Bowtie2 (Version 2.2.8) was used to compare short fragments to the ribosomal RNA (rRNA) database. The rRNA mapping read is then removed. The remaining clean reads are further used for assembly and gene abundance calculation. To establish the reference index of the genome and using HISAT2-2.2.4 to clean reads mapped to a reference genome (https://www.ncbi.nlm.nih.gov/assembly/GCF_000002315.6), the other parameter is set to the default. Then, RNA differential expression analysis was performed by DESeq2 (Love et al., 2014) software between two different groups (and by edgeR (Smyth, 2010) between two samples). The genes/transcripts with the parameter of false discovery rate (FDR) below 0.05 and an absolute fold change ≥2 were considered differentially expressed genes/transcripts.

#### 2.2.3 Gene Ontology and Kyoto Encyclopedia of Genes and Genomes Enrichment

Gene Ontology (GO) (Ashburner, 2000) is an international standardized gene functional classification system that offers a dynamic-updated controlled vocabulary and a strictly defined concept to comprehensively describe the properties of genes and their products in any organism. GO has three ontologies: molecular function, cellular component, and biological process. Genes usually interact with each other to play roles in certain biological functions. Pathway-based analysis helps further understand gene biological functions. Kyoto Encyclopedia of Genes and Genomes (KEGG) (Marisa, 2013) is the major public pathway-related database. Pathway enrichment analysis identified significantly enriched metabolic pathways or signal transduction pathways in DEGs compared with the whole genome background.

#### 2.2.4 Weighted Gene Co-Expression Network Analysis Analysis

WGCNA (weighted gene co-expression network analysis) is a systems biology method for describing the correlation patterns among genes across multiple samples. This method finds clusters (modules) of highly correlated genes and relates modules to external sample traits. The gene co-expression network was constructed using the R package WGCNA (Shannon et al., 2003) to identify modules of highly correlated genes based on the filtering data (mean expression level ≥1 and coefficient of variation ≥0.1). The core co-expression modules were visualized using Cytoscape_v3.8.2.

### 2.3 Metabolome Analysis

#### 2.3.1 Extraction and Detection of Metabolites

First, the samples were freeze-dried in accordance with the same proportion. Then, 1000 uL methanol (−20°C) redissolved lyophilized powder was transferred to a 2-ml centrifuge tube, followed by vortex oscillation for 1 min, and centrifugation at 12,000 rpm at 4°C for 10 min. 450 μL of the supernatant was taken in a 2-ml centrifuge tube and concentrated by a vacuum concentrator until dry. Then, 20 μL was taken from each sample to be tested and mixed into QC samples (QC: quality control, used to correct the deviation of the analysis result of the mixed sample and the error caused by the analysis instrument itself), and the remaining samples were used to be tested for LC-MS detection (Zelena et al., 2009; Dunn et al., 2018).

In chromatographic tests, chromatographic separation was accomplished in a Thermo Ultimate 3,000 system equipped with an ACQUITY UPLC® HSS T3 (150 × 2.1 mm, 1.8 µm, Waters) column maintained at 40°C. The temperature of the autosampler was 8°C. Gradient elution of analytes was carried out with (A) 0.1% formic acid in water and (B) 0.1% formic acid in acetonitrile or (C) 5 mM ammonium formate in water and (D) acetonitrile at a flow rate of 0.25 ml/min. Injection of 2 μL of each sample was administered after equilibration. An increasing linear gradient of solvent B (v/v) was used as follows: 0–1 min, 2% B/D; 1–9 min, 2–50% B/D; 9–12 min, 50–98% B/D; 12–13.5 min, 98% B/D; 13.5–14 min, 98–2% B/D; 14–20 min, 2% D-positive model (14–17 min, 2% B-negative model).

In mass spectrometry, the ESI-MSn experiments were executed on the Thermo Q Exactive mass spectrometer with the spray voltage of 3.8 kV and −2.5 kV in positive and negative modes, respectively. The sheath gas and auxiliary gas were set at 30 and 10 arbitrary units, respectively. The capillary temperature was 325°C. The analyzer scanned over a mass range of m/z 81-1 000 for a full scan at a mass resolution of 70,000. Data-dependent acquisition (DDA) MS/MS experiments were performed with an HCD scan. The normalized collision energy was 30 eV. Dynamic exclusion was implemented to remove some unnecessary information in MS/MS spectra.

#### 2.3.2 Data Processing and Metabolite Identification

The format of raw data files was converted into mzXML format using Proteowizard (v3.0.8789). Using R (v3.3.2) package XCMS (Want, 2006) to perform peak identification, peak filtration, peak alignment for each metabolite, the main parameters were set as follows: bw = 5, ppm = 15, peak width = c (5, 30), mzwid = 0.01, mzdiff = 0.01, method = “centWave”. Then, mass-to-charge ratio (m/z), retention time and intensity, and positive and negative precursor molecules were used for subsequent analysis. The peak intensities were batch-normalized to the total spectral intensity. The identification of metabolites is based on the exact molecular formula (molecular formula error <20 ppm). Then, peaks were matched with the Metlin (http://metlin.scripps.edu) and MoNA(https://mona.fiehnlab.ucdavis.edu//) to confirm annotations for metabolites.

### 2.4 Trend Analysis

Genes and metabolites expression pattern analysis is used to cluster metabolites of similar expression patterns for multiple samples (at least three in a specific time point, space, or treatment dose size order). To examine the expression pattern of all annotated genes and metabolites, the expression data of each sample (in the order of treatment) were normalized to 0, log2 (v1/v0), and log2 (v2/v0) and then clustered by Short Time-series Expression Miner software (STEM, version 1.3.11) (Ernst and Bar-Joseph, 2006).

The parameters were set as follows:

1) Maximum unit change in model profiles between time points is 1;
2) Maximum output profile number is 20 (similar profiles will be merged).
3) Minimum ratio of the fold change of DEGs is no less than 2.0.

The clustered profiles with a *p*-value ≤ 0.05 were considered significant profiles. Then, the genes and metabolites in all or each profile were subjected to KEGG pathway enrichment analysis. Through the hypothesis test of the *p*-value calculation and FDR (Benjamini and Hochberg, 1995) correction, the pathways with Q_value ≤ 0.05 were defined as significantly enriched pathways.

### 2.5 Integrated Analysis of the Transcriptome and Metabolome

Transcriptome and metabolome data were used to characterize the differences in gene expression and metabolite levels (Bylesjö et al., 2010). However, transcription and metabolism do not occur independently in biological systems. In order to reveal the regulatory influence mechanism between gene expression and metabolites during starvation-induced IM, the association analysis was carried out based on the same or similar change rules of genes or metabolites involved in the same biological process (Csardi and Nepusz, 2006; Kolde, 2015; Bouhaddani et al., 2016). The co-expression network between differential genes and metabolites in lipid metabolism was constructed using Cytoscape_v3.8.2.

## 3 Results

### 3.1 Transcriptome Analysis of the Liver of Laying Hens

In this study, we established 18 cDNA libraries with the following designations, RNA-seq generated 44, 786, and 614 to 95, 424, and 152 raw reads for each library. After filtering the low-quality reads, the average number of clean reads was 48, 911, and 475 (99.37%); 69,071, and 206 (99.40%); 62, 071, and 382 (99.40%); 55, 411, and 170 (99.36%); 55, 481, and 584 (99.28%); and 49, 951, and 232 (99.34%) for the F0F0, F3F3, F16F16, R6R6, R16R16, and R32R32 groups, respectively (Supplementary Table S1). The clean reads were used for all further analyses, and from them 91.51–92.91% of clean tags from the RNA-seq data mapped uniquely to the genome, while a small proportion of them (<2.93%) were mapped multiple times to the genome (Supplementary Table S2).

**FIGURE 1:**
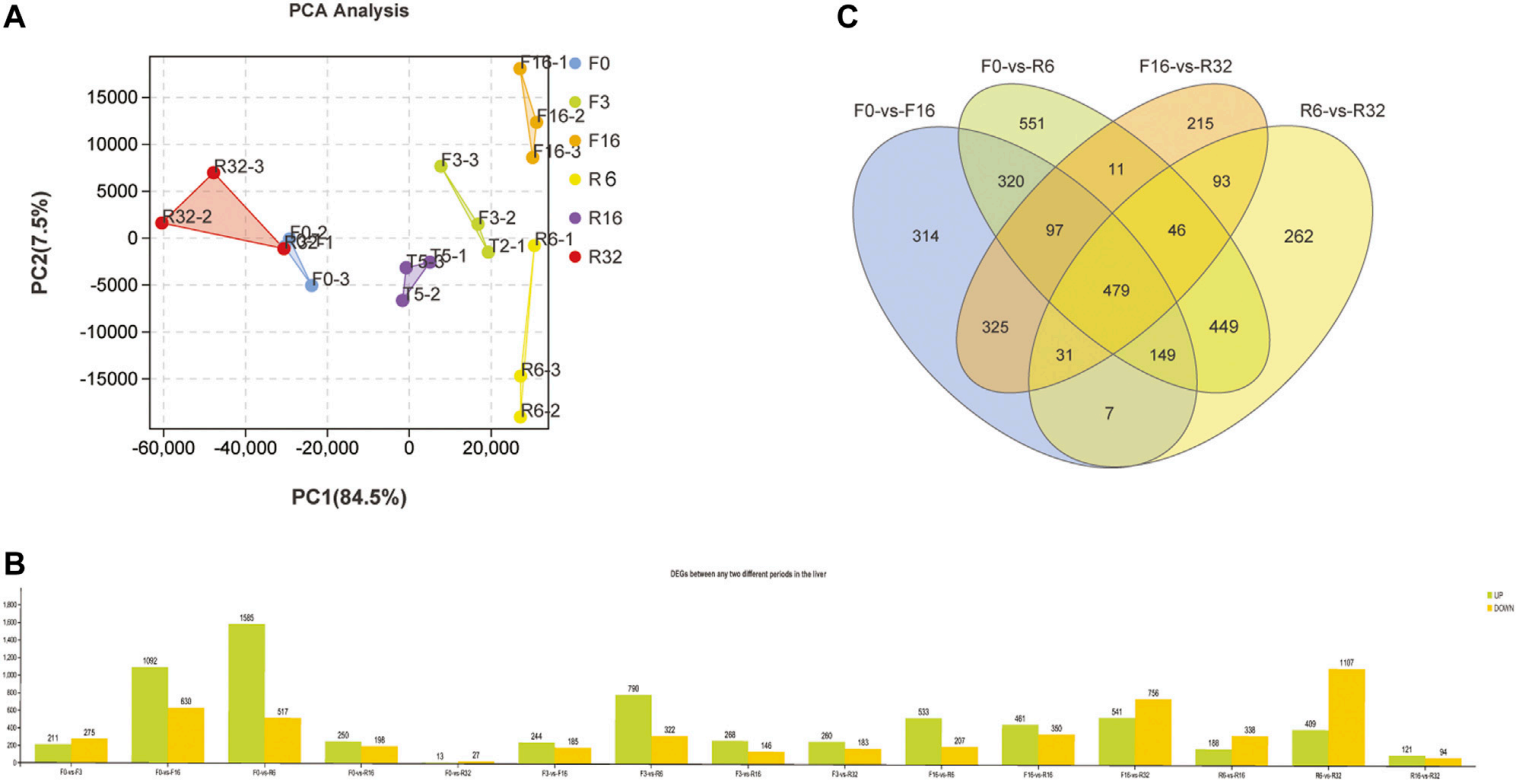
Transcriptome analysis of the liver in six periods during IM. **(A)** PCA analysis was used to understand the repeatability of samples in each period of IM. **(B)** Overall statistics of significantly different genes in each comparison group: green represents upregulation; yellow represents downregulation. **(C)** Difference Venn diagram of the relative comparison group.

To demonstrate the source of variance in our data, PCA analysis with two principal components (PC1 and 2) was performed. As shown in Figure 1A, PC score plots showed that the contribution of PC1 and 2 was 84.5% and 7.5%, respectively. The three individual samples collected at each time point were clustered closely together which validated the finding of low variance in the present analysis study and showed that the data could be used for the following analysis.

### 3.2 Differential Gene Expression in the Liver at Different Stages

FPKM was used to estimate the level of gene expression, and DEGSeq was used to examine the differential gene expression profile. The results showed that F0-VS-R32 and R16-VS-R32 had fewer differentially expressed genes, while F0-VS-F16, F0-VS-R6, F16-VS-R32, and R6-VS-R32 groups had more differentially expressed genes. Therefore, in order to further explore the dynamic gene expression pattern during the IM of laying hens, we conducted a study on DEGs in the F0-VS-F16, F0-VS-R6, F16-VS-R32, and R6-VS-R32 groups (Figure 1B). The Venn diagram shows the distribution of DEGs in the liver into four groups with 479 genes shared among the four groups (Figures 1C–F).

### 3.3 Gene Ontology Enrichment and Kyoto Encyclopedia of Genes and Genomes Pathway Analysis of DEGs Among the Four Groups

All DEGs in the four groups (F0-VS-F16, F0-VS-R6, F16-VS-R32, and R6-VS-R32) were analyzed using GO term enrichment and KEGG pathway. To investigate the significant pathways and related biological functions of DEGs during IM.

**FIGURE 2:**
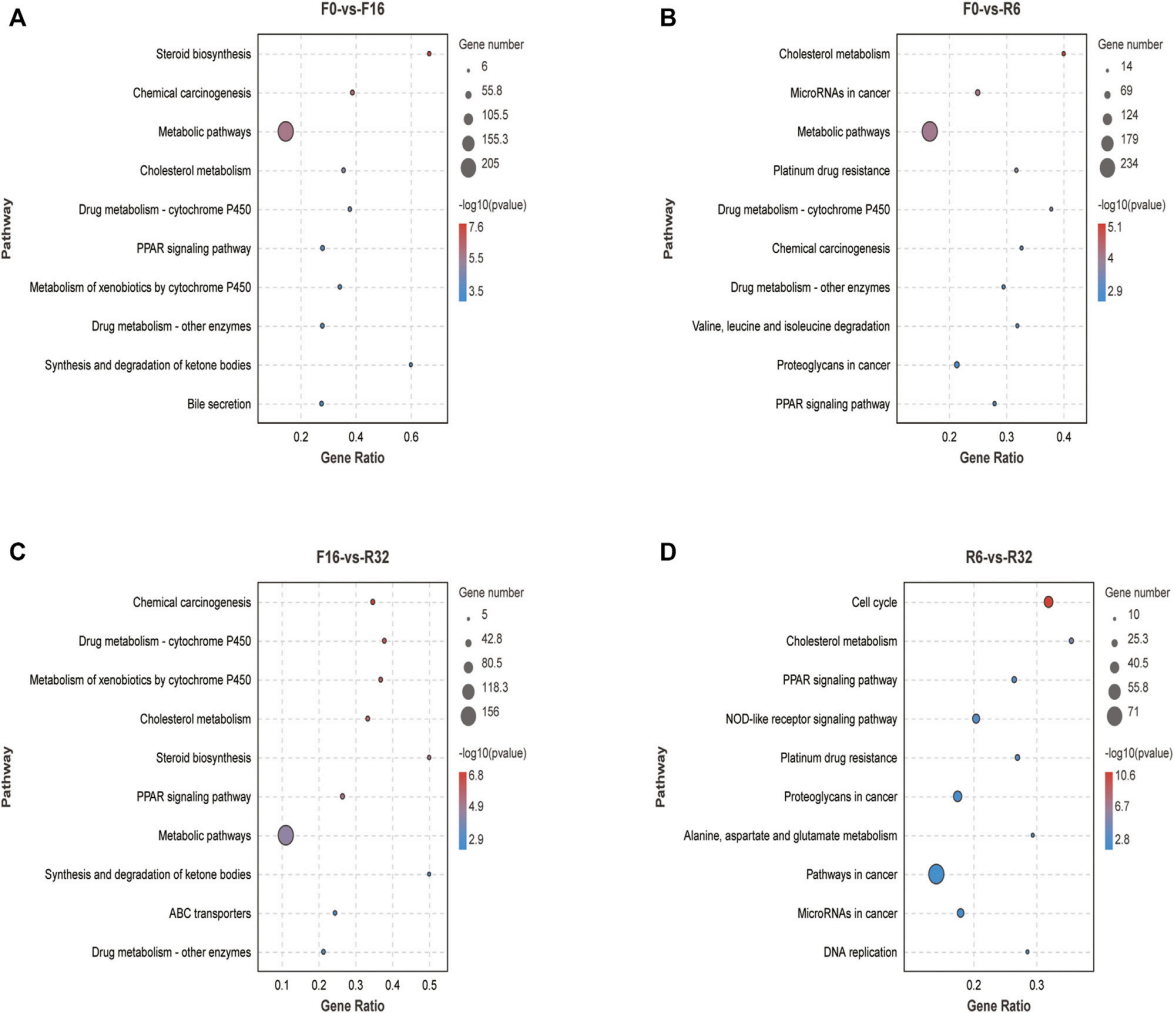
KEGG enrichment analysis of different genes in comparison groups during IM. **(A)** F0-vs-F16. **(B)** F0-vs-R6. **(C)** F16-vs-R32. **(D)** R6-vs-R32.

In our study, a total of 1722 DEGs from the F0-vs-F16 group in the liver were used for GO term enrichment (Supplementary Figure S1) and KEGG analyses (Figure 2A). We selected ten pathways (*p* < 0.05) from GO and KEGG and analyzed them. The GO term was mainly enriched in some pathways related to lipid metabolism, such as lipid metabolic process, lipid localization, lipid homeostasis, lipid biosynthetic process, sterol metabolic process, cholesterol homeostasis, sterol homeostasis, cholesterol metabolic process, and lipid transport. In addition, it was also enriched in the cellular response to chemical stimuli. KEGG was also enriched in some pathways related to lipid metabolism, such as steroid biosynthesis, cholesterol metabolism, synthesis, and degradation of ketone bodies. In addition, there were also important pathways such as the PPAR signaling pathway, drug metabolism—other enzymes, and bile secretion.

In the F0-vs-R6 group, a total of 2,102 DEGs in the liver were used to perform GO term (Supplementary Figure S2) and KEGG pathway analyses (Figure 2B). GO terms (*p* < 0.05) were enriched in cellular response to chemical stimulus, immune system process, and cytokine production, and KEGG was mainly enriched in cholesterol metabolism; valine, leucine, and isoleucine degradation; metabolic pathways; and proteoglycans in cancer.

A total of 1,297 DEGs from the liver of the F16-vs-R32 group were used to perform GO term (Supplementary Figure S3). and KEGG pathway analyses (Figure 2C). In GO term (*p* < 0.05), most pathways were related to lipid metabolism, including the lipid metabolic process, sterol metabolic process, lipid biosynthetic process, and lipid homeostasis. In addition, there were immune-related pathways, such as the immune system process and regulation of immune system process. The significant KEGG pathways were chemical carcinogenesis, drug metabolism—cytochrome P450, metabolism of xenobiotics by cytochrome P450, cholesterol metabolism, steroid biosynthesis, synthesis, and degradation of ketone bodies.

In the R6-vs-R32 group, the DEGs are mainly enriched in mitotic cell cycle, cell cycle, cell activation, cell cycle process, and cytokine production in GO term analysis (Supplementary Figure S4). Moreover, two immune-related pathways were also significant. In KEGG analysis (*p* < 0.05) (Figure 2D), cell cycle, cholesterol metabolism, pathways in cancer, and DNA replication were considered significant.

### 3.4 Co-Expression Network Analysis With Weighted Gene Co-Expression Network Analysis

**FIGURE 3:**
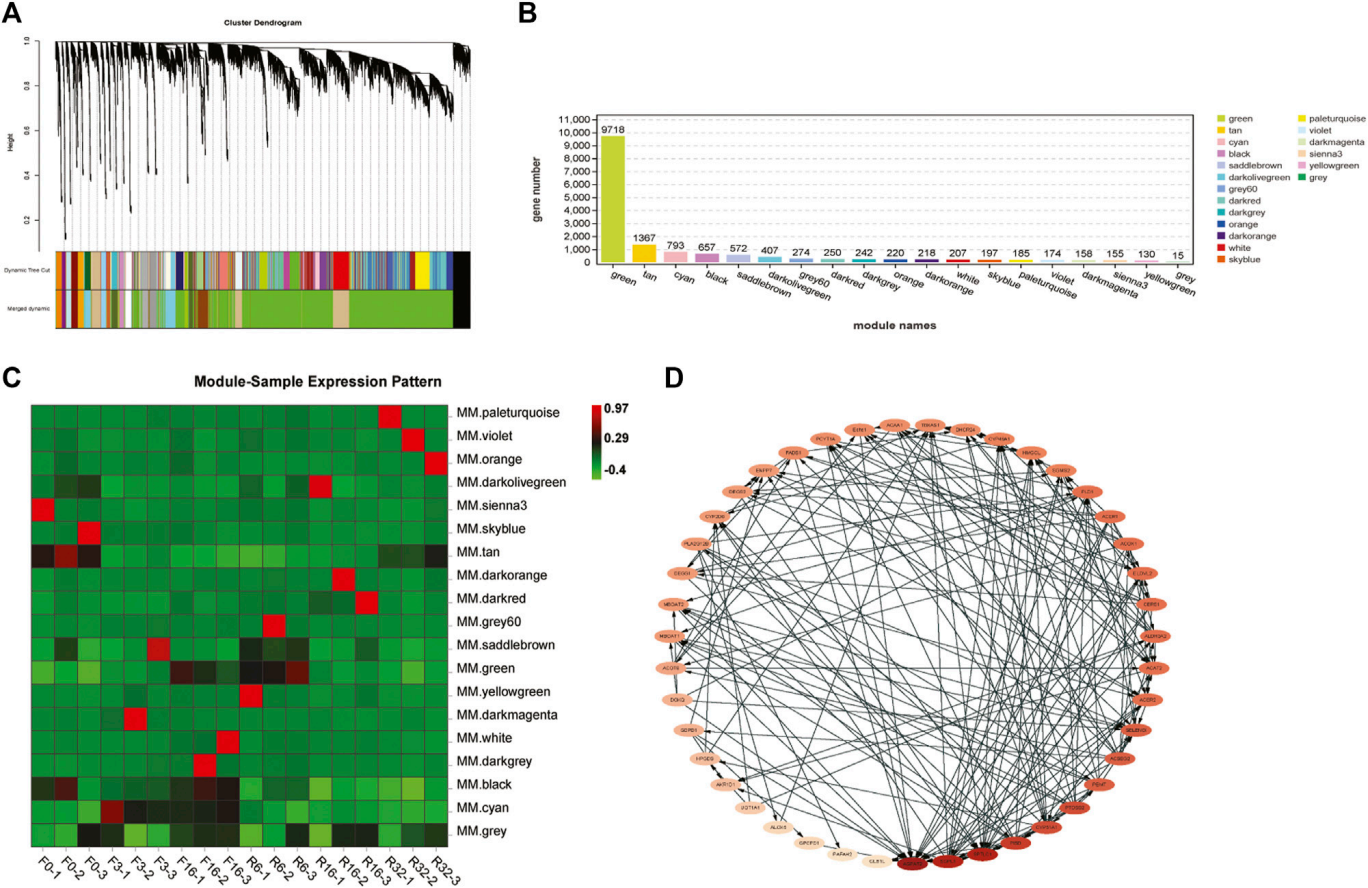
WGCNA of genes in the liver. **(A)** Hierarchical cluster tree showing coexpression modules identified by WGCNA. Each leaf in the tree represents one gene. The major tree branches constitute 19 modules labeled with different colors. **(B)** Number of genes contained in each module. **(C)** Expression patterns of module genes in each sample are displayed by module eigenvalues. The value of module eigenvalues in each sample reflects the comprehensive expression level of all genes in each sample. **(D)** In selected modules, genes related to lipid metabolism interact with each other in the co-expression network, and the darker the color, the stronger the connectivity.

Between genes have mutual induction and deter expression or synergy; these effects will result in the expression of related gene correlation between the amount, in the case of a large sample, the classification of gene expression was conducted more regularly, In this study, tens of thousands of genes were divided into 19 modules (color-coded) using WGCNA analysis with similar expression patterns, shown by the dendrogram (Figure 3A; Supplementary Figure S5), in which each tree branch constitutes a module, and each leaf in the branch is one gene. Due to the time-specific expression profile of the characteristic genes, 19 modular characteristic genes from 19 different modules were associated with different types of IM periods (Figures 3B, C). Through Figure 3C, we found MM. tan, MM. green, and MM. cyan modules that are significantly correlated with specific samples so that corresponding modules can be selected for further research (the module eigenvalue is equivalent to the weighted composite value of all gene expression levels in the module).

KEGG enrichment analysis was conducted for these three modules, focusing only on the lipid metabolism pathway, and then all genes (including genes with significant differences and genes with no significant differences) in the lipid metabolism pathway were selected for network interaction analysis, with the purpose of discovering those key genes neglected due to the transient expression. As shown in Figure 3D, a total of 43 genes were obtained through interaction. Using Cytoscape software, the connectivity of each gene was calculated. Generally, genes with high connectivity are regarded as hub genes. In the interaction network, the color of the gene gradually deepened as connectivity increased. Among them, AGPAT2F3, SGPL1, SPTLC1, PISD, and CYP51A1 are considered to have high connectivity and are the key genes in the network.

**FIGURE 4:**
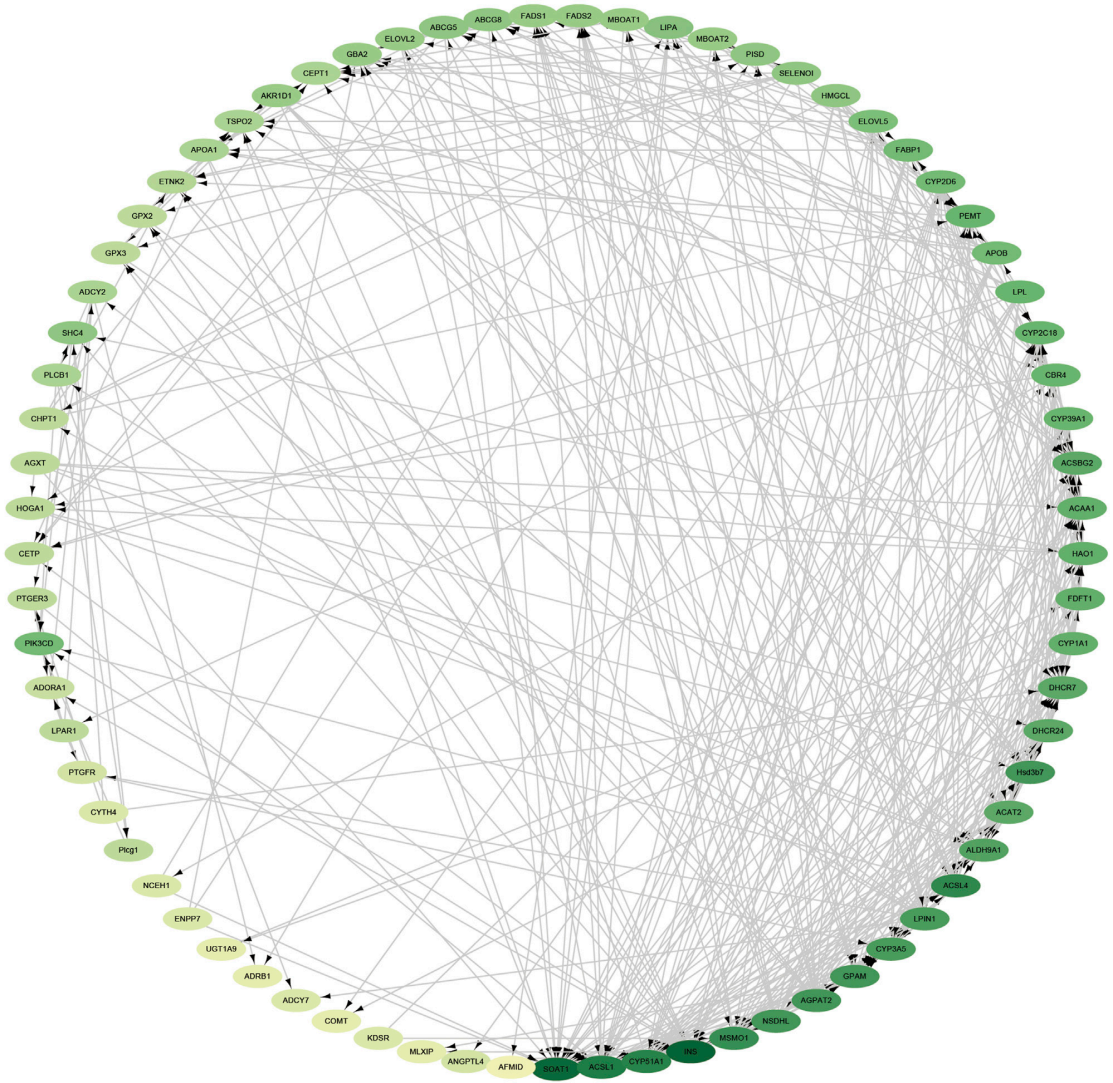
WGCNA of genes associated with lipid metabolism. co-expression network interaction of all lipid metabolism–related genes [including genes derived from module analysis and genes in lipid metabolism-related pathways (did not require *p* < 0.05)], and the darker the color, the stronger the connectivity.

Then, we conducted co-expression network analysis between the selected genes in the module analysis and all the genes involved in lipid metabolism in the IM process (Figure 4) so as to dig out more potential core genes, which may have little difference in expression but are consistent with the expression trend of these different genes. We chose the top 10 genes; they were INS, SOAT1, ACSL1, CYP51A1, ACSL4, MSMO1, AGPAT2, Hsd3b7, GPAM, and NSDHL.

**FIGURE 5:**
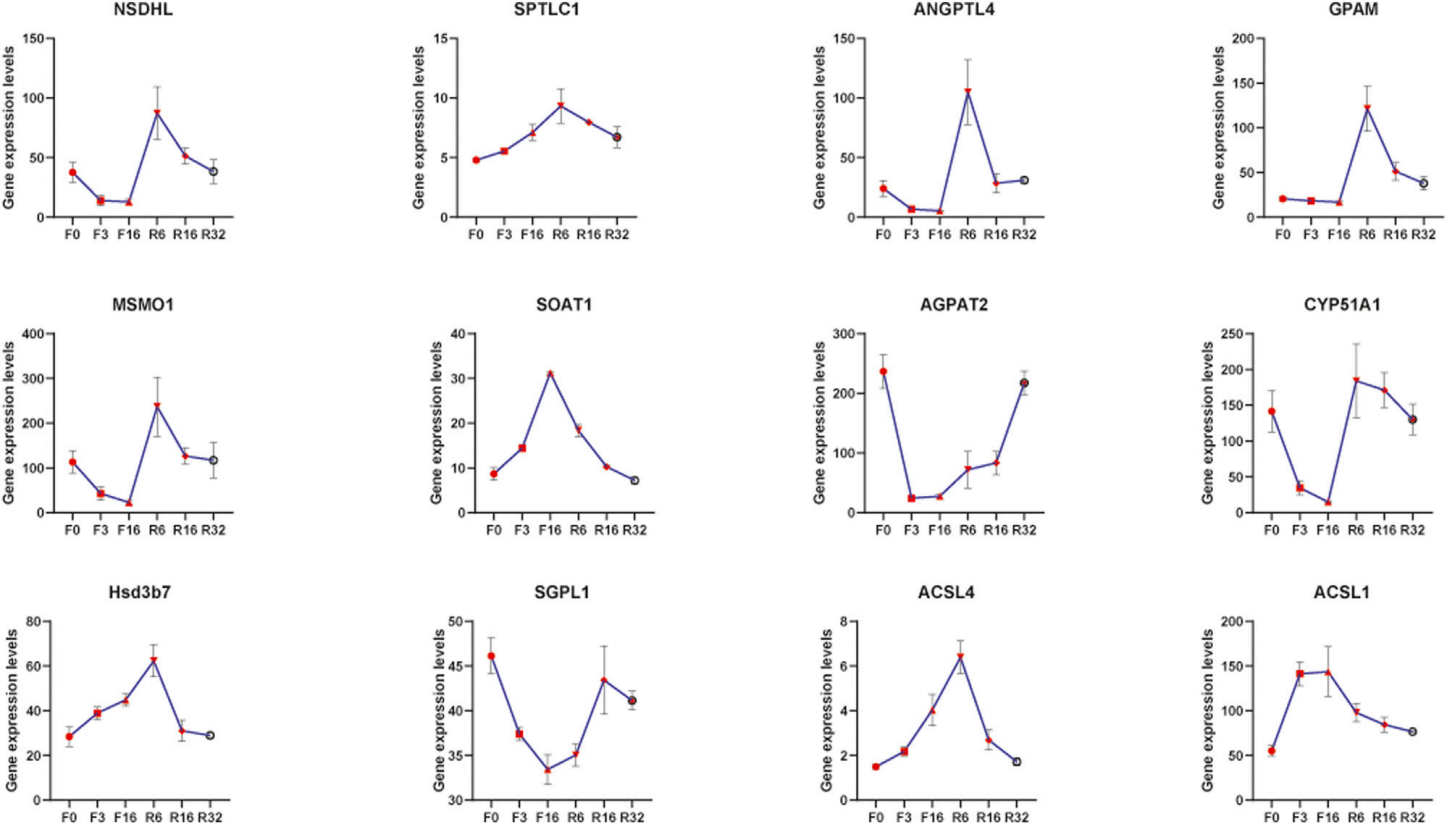
Expression trends of genes related to lipid metabolism during IM.

Transcriptome data showed significant changes in genes involved in lipid metabolism pathways in the liver of laying hens during IM, and the expression trends of these genes were similar to some extent (Figure 5). The expression of some genes (AGPAT2, SGPL1, PISD, CYP51A1, MSMO1, GPAM, and NSDHL) decreased gradually during starvation, with the degree of downregulation of these genes increasing as starvation time extended and gradually returning to their pre-experiment levels when feeding resumed. On the contrary, the expression levels of other genes (SPTLC1, SOAT1, ACSL1, ACSL4, and HSD3B7) were increasingly upregulated with the extension of starvation time and decreased to pre-experiment levels after resuming feeding for a period of time.

### 3.5 Metabolomics Profiling

Based on the transcriptome results, we selected five important stages (F0, F3, F16, R6, and R32) for cecal content metabolome sequencing of laying hens. Based on the transcriptome results, we selected five important stages (F0, F3, F16, R6, and R32) for cecal content metabolome sequencing of laying hens. The ionization source of LC/MS was electrospray ionization, which included positive (POS) and negative (NEG) ion modes. The QC samples were analyzed to detect the stability and repeatability of the system. The peak retention time (RT) and peak area of total ion chromatograms from all QC samples overlapped well, thereby indicating that the analytical system was stable (Supplementary Figure S6). A total of 2016 and 1,597 valid peaks were identified in the POS and the NEG modes, respectively, in metabolomics and matched 1781 (POS) and 1,448 (NEG) metabolites, respectively, in the metabolome based on the in-house MS2 database.

**FIGURE 6:**
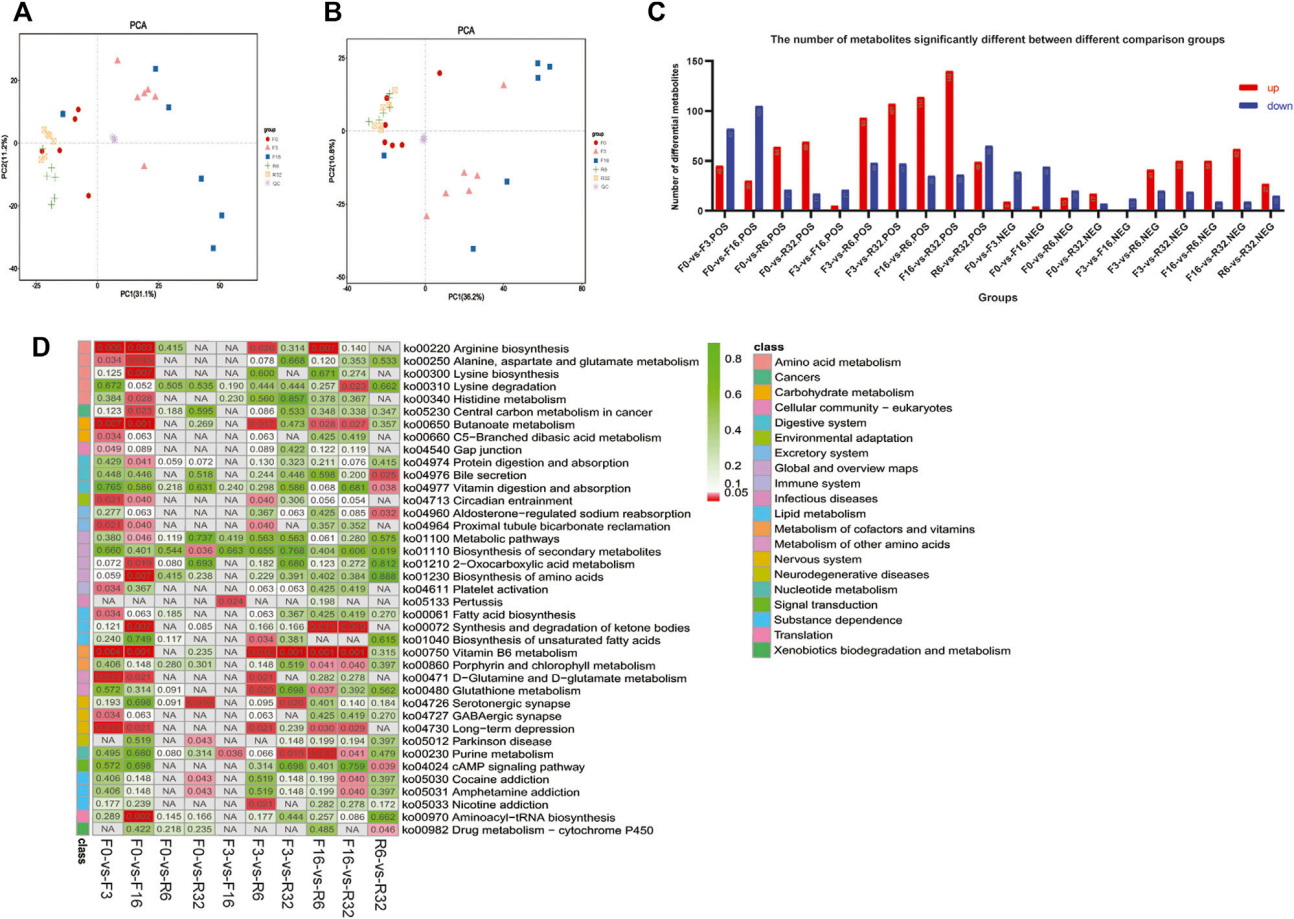
Metabolome analysis. Principal component analysis (PCA) was performed on all samples and quality control samples in **(A)** positive ion mode and **(B)** negative ion mode. **(C)** Number of differential metabolites between the comparison groups in positive and negative ion modes. **(D)** KEGG enrichment analysis of differential metabolites.

Principal component analysis (PCA) was performed on all samples and QC samples (Figures 6A, B), and the stability and reliability of instrumental analysis could be obtained by observing the dispersion between QC samples. Orthogonal least partial square discriminant analysis (OPLS-DA) is a derivative algorithm of PLS-DA. Compared with PLS-DA, OPLS-DA combines two methods of orthogonal signal correction (OSC) and PLS-DA, which can decompose the X matrix information into two types of information related to Y and irrelevant information. By removing the irrelevant differences, the relevant information is concentrated in the first predictive component. The OPLS-DA results were used to analyze subsequent model tests and differential metabolite screening (Supplementary Figure S7).

### 3.6 Differential Metabolite Screening

We combined the VIP value of multivariate statistical analysis OPLS-DA and the *t*-test *p*-value of univariate statistical analysis to screen the significantly differential metabolites between different comparison groups (Saccenti et al., 2014). The threshold for significant difference was VIP≥1 and *t*-test (*p* < 0.05). The metabolite results with the significant differences are shown in (Figure 6C).

Subsequently, KEGG enrichment analysis (Figure 6D) was conducted for the differential metabolites, and the results showed that the differential metabolites were mainly enriched in amino acid metabolism, cancers, carbohydrate metabolism, cellular community eukaryotes, digestive system, environmental adaptation, excretory system, global and overview maps, immune system, lipid metabolism, and nervous system.

### 3.7 Integrated Analysis of the Transcriptome and Metabolome

**FIGURE 7:**
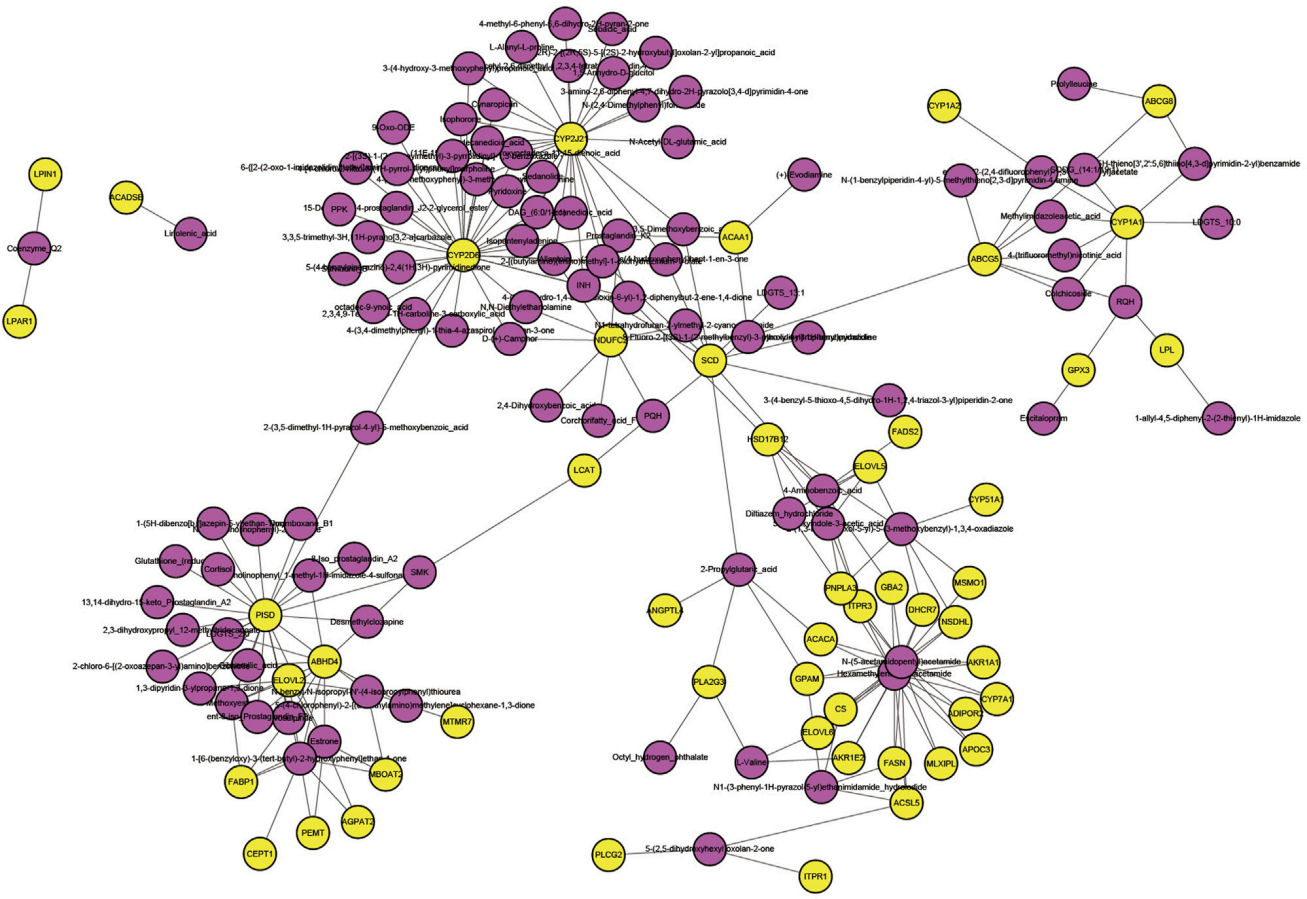
Network interactions of key genes and differential metabolites; yellow represents genes and pink represents metabolites.

We correlated all genes associated with lipid metabolism (genes with significant and nonsignificant differences) with metabolome data. As you can see (Figure 7), yellow represents genes and pink represents metabolites. The correlations between these genes and metabolites were all greater than 0.88 (both positive and negative). We correlated all genes associated with lipid metabolism (genes with significant and nonsignificant differences) with metabolome data. We analyzed the connectivity of these genes and metabolites, and the top 20 were CYP2D6, CYP2J21, PISD, N-(5-acetamidopentyl) acetamide, hexamethylene bisacetamide, ABHD4, NDUFC2, SCD, 1-[6-(benzyloxy)-3-(tert-butyl)-2-hydroxyphenyl]Ethan1-one, CYP1A1, estrone, 2-(1,3-benzodioxol-5-yl)-5-(3-methoxybenzyl)-1,3,4-oxadiazole, 4-aminobenzoic_acid, 2-propylglutaric acid, ELOVL2, HSD17B12, PNPLA3, RQH, 5-fluoro-2-[(3S)-1-(2-methylbenzyl)-3-pyrrolidinyl]-1H-benzimidazole, and 2-methoxyestrone (the variation trend of these genes and metabolites during IM is described in Supplementary Materials).

## 4 Discussion

### 4.1 Induced Molting can Improve the Performance of Laying Hens in the Next Laying Stage by Regulating Lipid Metabolism

Hunger is a physiological imbalance caused by lack of food or nutrition in the body. When the glucose level in the blood drops to the range of hypoglycemia, the body’s compensation mechanism will be activated (Staehr et al., 2004). Hunger will increase the production of non-esterified fatty acids (NEFA) in adipose tissue and start the fat mobilization mechanism (Ensling et al., 2011). As an energy substance, fat has many advantages compared with other macromolecules. For example, fat can be stored in adipose tissue in the form of low water content and high energy density. The amount of fat in the body also varies widely (Lindström, 1991; Castellini and Rea, 1992). Lipolysis mainly includes the hydrolysis of triglycerides and the oxidation of fatty acids, in which the hydrolysis of triglycerides into fatty acids and glycerol happens under the joint action of triglyceride lipase, hormone-sensitive esterase, and lipoprotein esterase. Fatty acid oxidation is the formation of fatty acid esters coA under the action of esters coA synthase (ACSL) (Castellini and Rea, 1992). During IM, laying hens experienced long periods of starvation, and in the absence of external energy supplies, the hens used stored body fat to obtain energy; the expression levels of ACSL1 and ACSL4 were significantly upregulated during starvation.

**FIGURE 8:**
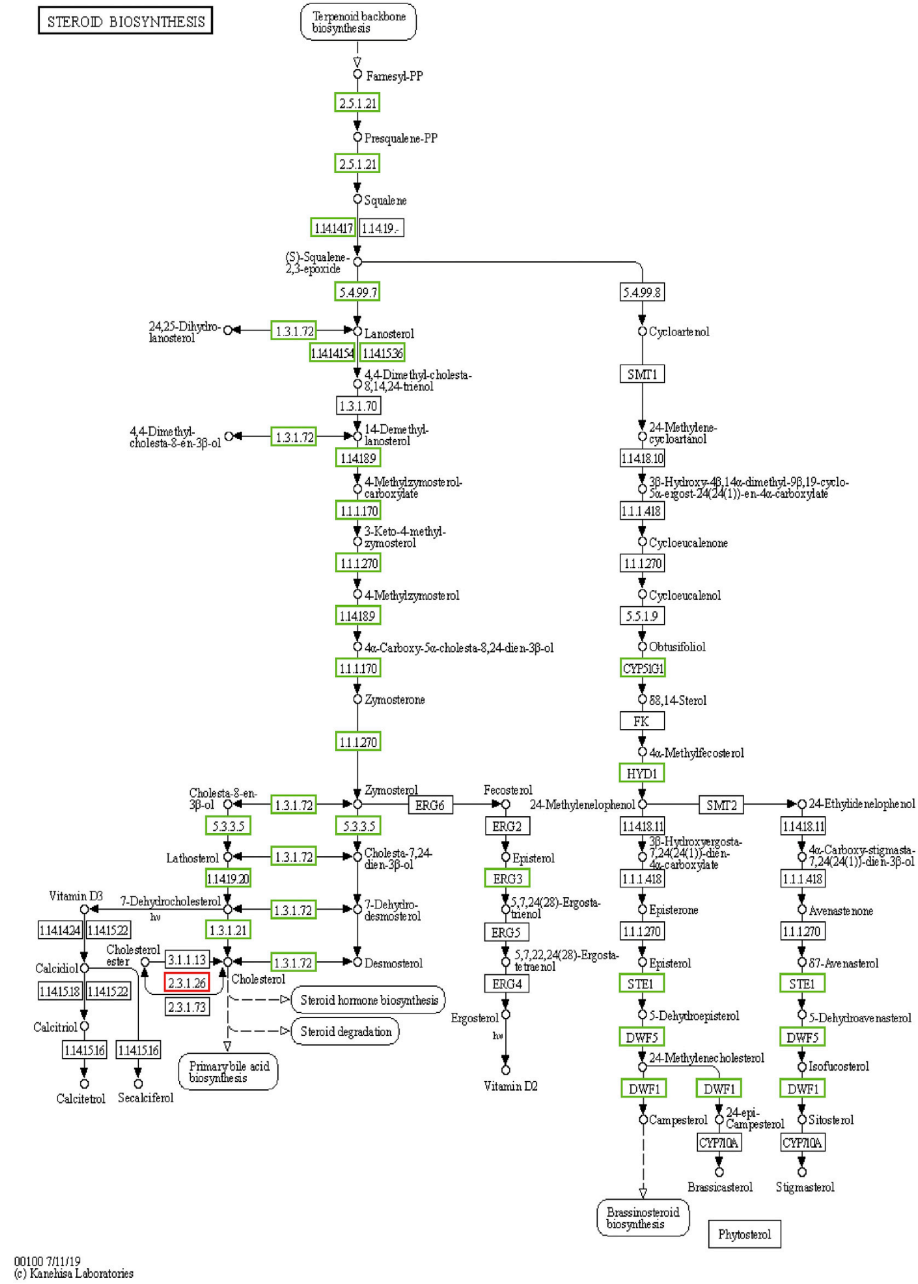
Multiple genes in the F0-VS-F16 steroid biosynthesis pathway were significantly downregulated during IM.

It is well known that lipid metabolism and transport in the liver are closely related to the laying performance of laying hens (Liu et al., 2018). Cholesterol plays an important role in lipid metabolism. CYP51A1 (sterol 14alpha-demethylase) is a late regulator of cholesterol synthesis (Kojima et al., 2000; Degawa, 2006). In this study, the expression level of CYP51A1 decreased significantly in F3 and F16, which is due to the loss of energy supply and the lack of precursor substances in cholesterol synthesis of laying hens. After the energy supply was restored, CYP51A1 expression was significantly upregulated. The expression trend of MSMO1 (methylsterol monooxygenase) and NSDHL (sterol-4alpha-carboxylate 3-dehydrogenase) in the same pathway as CYP51A1 is similar to that of CYP51A1. After IM, the expression level of genes in the steroid biosynthesis pathway was downregulated and increased to the pre-experiment level and tended to exceed the pre-experiment gene expression level (Figure 8). Importantly, cholesterol is a precursor to estrogen (the steroid hormone). These results indicated that IM increased the laying rate of laying hens in the second laying cycle at the mRNA level, which was worthy of affirmation.

In addition, PISDs (phosphatidylserine decarboxylases), also known as phosphatidylserine decarboxylase, comprise pyridoxal phosphate and pyruvate. It is a key enzyme in the glycerophospholipid metabolism pathway (Marescaux, 2007). At F3 and F16, the expression of the PISD gene was significantly downregulated. The expression levels of PTDSS2, SELENOI, PEMT, PLD1, PLA2G12B, DGKQ, GPCPD1, GPAM, CEPT1, LPIN1, MBOAT2, and AGPAT2in the same pathway of PISD also showed a similar trend in the starvation stage. This indicates that the body preferentially uses the products of fat mobilization for energy supply (Thouzeau et al., 1997), rather than the production of phospholipids, which protect and regulate metabolism under the condition of long-term starvation. The expression levels of these genes were significantly upregulated when feeding resumed, and the laying hens had enough energy in their bodies. Egg lecithin is a kind of compound phospholipid extracted from egg yolk mainly comprising phosphatidyl choline, PC); phosphatidyl ethanolamine, PE); phosphatidyl inositol, PI); and phasphotidyl serine (PS). From the expression of related genes in the glycerolipid pathway, we can also see that the glycerolipid pathway is significantly active during the recovery stage of IM, possibly in preparation for the formation of egg yolk in the next laying stage.

### 4.2 Effects of Intestinal Microflora on Laying Performance of Laying Hens Through the“Gut-Liver Axis”

There are a large number of relatively stable microbial communities in the digestive tract of poultry. They play a very important role in maintaining the relative stability of the poultry gastrointestinal tract and nutrient digestion and absorption (Jiangrang, 2003; Ilina, 2016). The cecum is the most developed site of intestinal microorganisms in poultry (Jianhua, 2010; Kang et al., 2021; Pedroso et al., 2021). However, studies have found that gut microbes form a mutualistic symbiosis with their hosts during a long process of coevolution. Gut microbes can sense changes in the intestinal environment of the host while obtaining nutrients needed for survival, change the gene expression of the host and their own, and establish a mutualistic relationship with the host (Isberg and Barnes, 2002). In human medicine, there is growing evidence that changes in human genetic background, diet, and antibiotic treatment can affect gut microbes, which in turn affect host metabolism (Goodrich et al., 2014; Ting, 2014; Zhang et al., 2015). At present, there are more and more studies on the regulation of intestinal flora on metabolic diseases in the human body. Gut microbiome composition, changes, and imbalances are closely related to host metabolism and can affect a variety of diseases including obesity, type 2 diabetes, and inflammatory bowel disease. In animals, intestinal microorganisms have been confirmed to be closely related to lipid deposition in mice, pigs, and poultry (Guo et al., 2008; Emmanouil et al., 2010; Guo et al., 2010).

Some studies (Pi et al., 2017) have shown that the body and its intestinal microbes can metabolize and produce some small molecules, such as phenols, SCFAs, and bile salts, which play a crucial role in the association of information between host cells and host symbiotic microorganisms and in the health of the body. The metabolites in the cecum are closely related to the life activities and material metabolism of the body.

**FIGURE 9:**
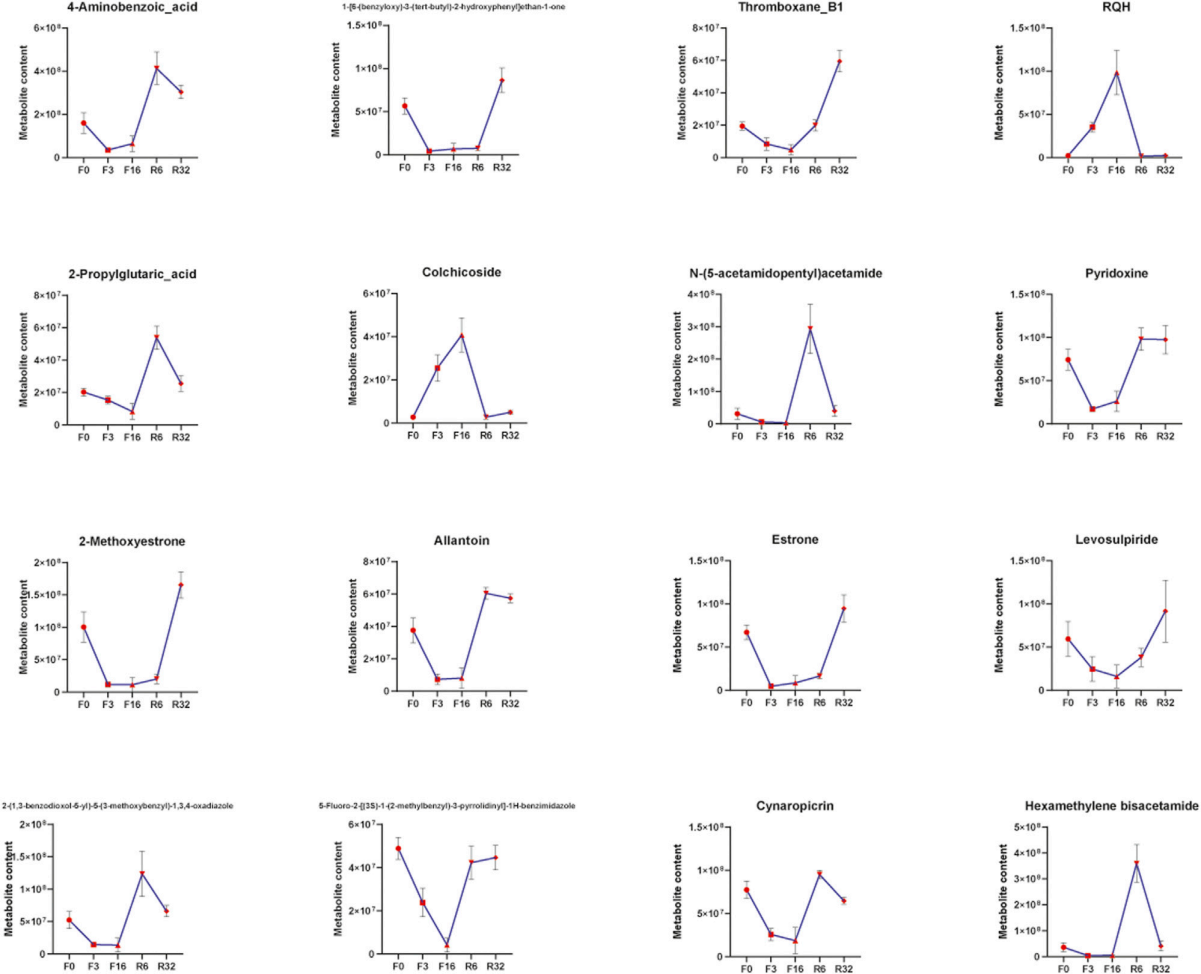
Changes in metabolite content during IM.

In this study, we also found significant changes in metabolites in the cecum (Figure 9). In our results, colchicine content increased significantly at F3 and F16 but decreased to the pre-experiment level after resuming feeding. In clinical medicine, colchicine is mainly used for the treatment of acute gout, but it has toxic side effects; colchicine can cause liver damage in rats. Colchicine inhibits the expression of nuclear receptor FXR, resulting in the imbalance of bile acid regulation in hepatocytes (Yan-Yan et al., 2018). In this study, the gene associated with colchicine was CYP1A1(cytochrome P450 family 1 subfamily A member 1), and the expression trend of this gene was similar to the change in colchicine content. Studies have shown that CYP1A1 is related to hormone and metabolism of a variety of exogenous toxic substances, such as benzopyrene and dioxins. Moreover, the expression of CYP1A1 gene polymorphism is closely related to the susceptibility of cervical cancer, prostate cancer, childhood acute leukemia, lung cancer, esophageal cancer, and other tumors (Nerurkar et al., 2000). At F3, in terms of the growth rate of colchicine and CYPA1, colchicine content increased faster than CYP1A1 expression. These results indicate that cecal metabolites do affect gene expression in the liver and further demonstrate that intestinal microorganisms and their metabolites play a regulatory role in the metabolic activities of the body.

Furthermore, microorganisms can also regulate lipid metabolism. Angiopoietin-like protein 4 (ANGPTL4) (Aimin, 2005; Alex et al., 2013), also known as fasting inducer factor (FIAF), is a protein closely related to fat metabolism in animals. Studies have shown that overexpression of ANGPTL4 can induce hepatic enlargement and fatty liver formation in mice (Aimin, 2005; Yi, 2016). Some scholars have found that intestinal microorganisms can regulate the expression of ANGPTL4 in intestinal epithelial cells directly or indirectly (metabolites) (Grootaert et al., 2011). Some researchers have also found that intestinal microbial metabolites have a regulatory effect on host ANGPTL4, thus affecting host lipid metabolism (Zhao et al., 2014). In this study, ANGPTL4 was downregulated at F3 and F16 and significantly upregulated and exceeded the expression level at F0 after resuming feeding. The cecal metabolite associated with ANGPTL4 is 2-propyl glutaric acid, and its content variation trend is related to ANGPTL4.

In addition, estrogen content decreased significantly at F3 and F16 and increased significantly at R6 and R16, higher than the level before the test, which was consistent with the expression trend of PISD, AGPAT2, MBOAT2, and PEMT. After 32 days of recovery, the level of estrogen in laying hens was much higher than the level before the experiment. After IM, the level of estrogen was regulated by increasing PISD, AGPAT2, MBOAT2, and PEMT gene expression to stimulate the laying performance of laying hens so as to enter a new reproductive cycle.

In conclusion, intestinal microbes are closely related to metabolic activities, especially lipid metabolism, of their hosts. However, lipid metabolism in poultry liver is closely related to laying performance. Therefore, it is reasonable to believe that microorganisms and their metabolites in the cecum of laying hens are related to laying performance.

## 5 Conclusion

During IM, laying hens had a great influence on the liver and gut, but as to the recovery of food intake, laying hens in the second cycle of egg production rate and egg quality show improvement, and our research results show that in the whole experiment process, laying hens in the cecum metabolites and genes in the liver do have interaction relations; however, whether this relationship is two-way interaction or one-way regulation remains to be studied, which also points out the direction for our next research.

## Data Availability

The original contributions presented in the study are publicly available. This data can be found here BioProject: PRJNA811637. The database connection: https://dataview.ncbi.nlm.nih.gov/object/PRJNA811637.
