# Supplementary material for: Interaction Between Cecal Metabolites and Liver Lipid Metabolism Pathways During Induced Molting in Laying Hens
Source: Front Physiol. 2022 May 20;13:862721. doi: 10.3389/fphys.2022.862721 (PMC9169092; doi:10.3389/fphys.2022.862721)
Supplement: Supplementary file 1 [file DataSheet1.docx]

**Supplementary information**

**Supplementary Table S1** Sequencing data filtering statistics of 18 liver samples

**Supplementary Table S2** DEGs between 15 groups in the liver

**Supplementary Figure S1** Significant GO enrichment of DGEs(P<0.05) in T1-vs-T3

**Supplementary Figure S2** Significant GO enrichment of DGEs(P<0.05) in T1-vs-T4

**Supplementary Figure S3** Significant GO enrichment of DGEs(P<0.05) in T3-vs-T6

**Supplementary Figure S4** Significant GO enrichment of DGEs(P<0.05) in T4-vs-T6

**Supplementary Figure S5** The Power value curve

**Supplementary Figure S6** QC samples are mixed by taking 20 µL from each sample to be tested, which is used to correct the deviation of mixed sample analysis results and errors caused by the analysis instrument itself.

**Supplementary Figure S7** The sorting inspection(POS). The sorting test is a random sorting

**Supplementary Figure S8** The sorting inspection(NEG). The sorting test is a random sorting

**Supplementary Table S1** Sequencing data filtering statistics of 18 liver samples

| **Sample** | **RawDatas** | **CleanData(%)** | **Adapter(%)** | **LowQuality(%)** | **polyA(%)** | **N(%)** |
| --- | --- | --- | --- | --- | --- | --- |
| T1-1 | 46426480 | 46107120 (99.31%) | 53266 (0.11%) | 247338 (0.53%) | 0 (0.00%) | 18756 (0.04%) |
| T1-2 | 44786614 | 44537636 (99.44%) | 46450 (0.10%) | 184490 (0.41%) | 0 (0.00%) | 18038 (0.04%) |
| T1-3 | 56456094 | 56095670 (99.36%) | 61380 (0.11%) | 276344 (0.49%) | 0 (0.00%) | 22700 (0.04%) |
| T2-1 | 56664368 | 56295976 (99.35%) | 70688 (0.12%) | 275254 (0.49%) | 0 (0.00%) | 22450 (0.04%) |
| T2-2 | 95424152 | 94942924 (99.50%) | 108916 (0.11%) | 372312 (0.39%) | 0 (0.00%) | 0 (0.00%) |
| T2-3 | 56298766 | 55977720 (99.43%) | 57916 (0.10%) | 240546 (0.43%) | 0 (0.00%) | 22584 (0.04%) |
| T3-1 | 62032952 | 61679684 (99.43%) | 57766 (0.09%) | 270656 (0.44%) | 0 (0.00%) | 24846 (0.04%) |
| T3-2 | 55215964 | 54881304 (99.39%) | 63516 (0.12%) | 248616 (0.45%) | 0 (0.00%) | 22528 (0.04%) |
| T3-3 | 70090576 | 69662160 (99.39%) | 90196 (0.13%) | 309898 (0.44%) | 0 (0.00%) | 28322 (0.04%) |
| T4-1 | 60017640 | 59641068 (99.37%) | 64114 (0.11%) | 288370 (0.48%) | 0 (0.00%) | 24088 (0.04%) |
| T4-2 | 56671626 | 56320350 (99.38%) | 69320 (0.12%) | 258986 (0.46%) | 0 (0.00%) | 22970 (0.04%) |
| T4-3 | 50607186 | 50269092 (99.33%) | 58840 (0.12%) | 258790 (0.51%) | 0 (0.00%) | 20464 (0.04%) |
| T5-1 | 54334618 | 53963124 (99.32%) | 59066 (0.11%) | 290794 (0.54%) | 0 (0.00%) | 21634 (0.04%) |
| T5-2 | 60436222 | 59979186 (99.24%) | 77526 (0.13%) | 354888 (0.59%) | 0 (0.00%) | 24622 (0.04%) |
| T5-3 | 52904984 | 52517442 (99.27%) | 66350 (0.13%) | 299884 (0.57%) | 0 (0.00%) | 21308 (0.04%) |
| T6-1 | 51814818 | 51498338 (99.39%) | 56314 (0.11%) | 239748 (0.46%) | 0 (0.00%) | 20418 (0.04%) |
| T6-2 | 51891310 | 51526508 (99.30%) | 61380 (0.12%) | 282312 (0.54%) | 0 (0.00%) | 21110 (0.04%) |
| T6-3 | 47144728 | 46834850 (99.34%) | 47786 (0.10%) | 242774 (0.51%) | 0 (0.00%) | 19318 (0.04%) |

**Supplementary Table S2** DEGs between 15 groups in the liver

| Period | Up-regulated genes | Down-regulated gene | Total |
| --- | --- | --- | --- |
| T1-vT-T2 | 211 | 275 | 486 |
| T1-vT-T3 | 1092 | 630 | 1722 |
| T1-vT-T4 | 1585 | 517 | 2102 |
| T1-vT-T5 | 250 | 198 | 448 |
| T1-vT-T6 | 13 | 27 | 40 |
| T2-vT-T3 | 244 | 185 | 429 |
| T2-vT-T4 | 790 | 322 | 1112 |
| T2-vT-T5 | 268 | 146 | 414 |
| T2-vT-T6 | 260 | 183 | 443 |
| T3-vT-T4 | 533 | 207 | 740 |
| T3-vT-T5 | 461 | 350 | 811 |
| T3-vT-T6 | 541 | 756 | 1297 |
| T4-vT-T5 | 188 | 338 | 526 |
| T4-vT-T6 | 409 | 1107 | 1516 |
| T5-vT-T6 | 121 | 94 | 215 |

**Supplementary Figure S1** Significant GO enrichment of DGEs(P<0.05) in T1-vs-T3


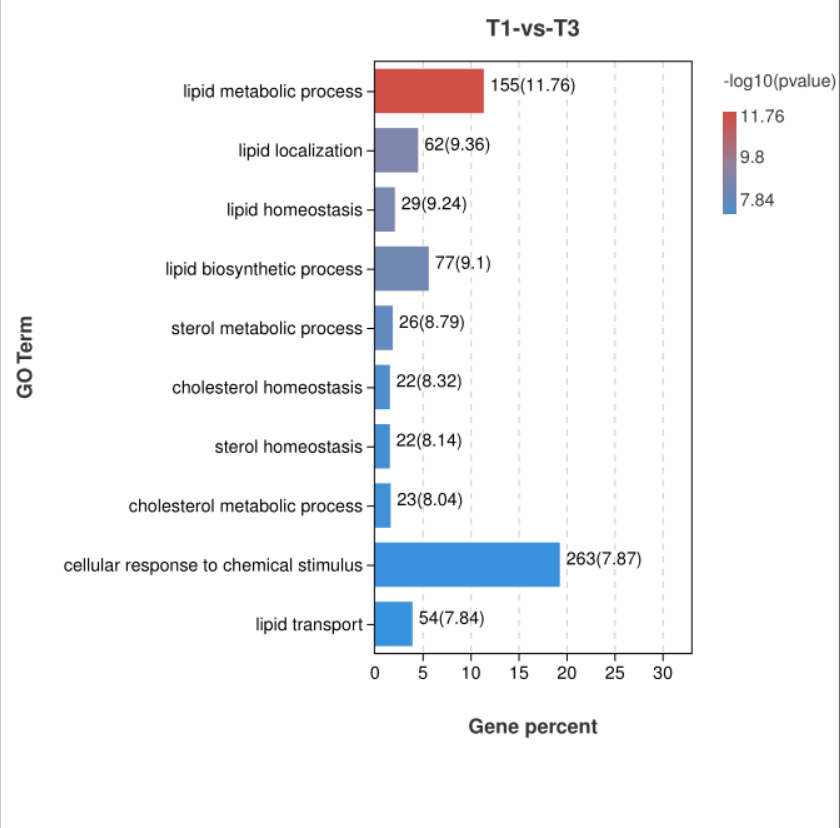


**Supplementary Figure S2** Significant GO enrichment of DGEs(P<0.05) in T1-vs-T4


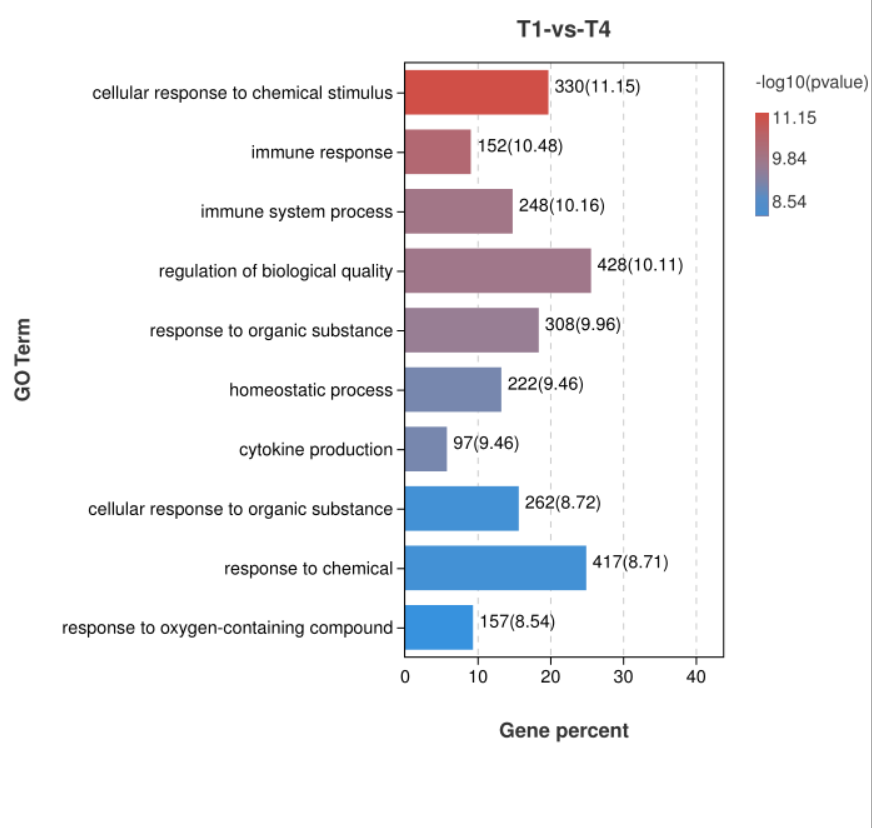


**Supplementary Figure S3** Significant GO enrichment of DGEs(P<0.05) in T3-vs-T6


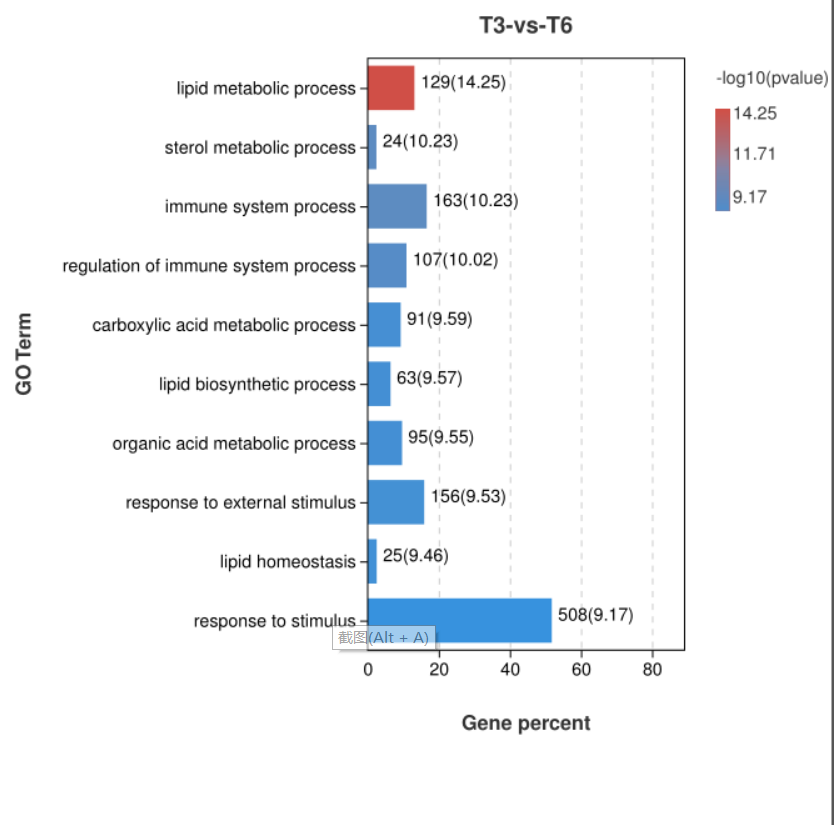


**Supplementary Figure S4** Significant GO enrichment of DGEs(P<0.05) in T4-vs-T6


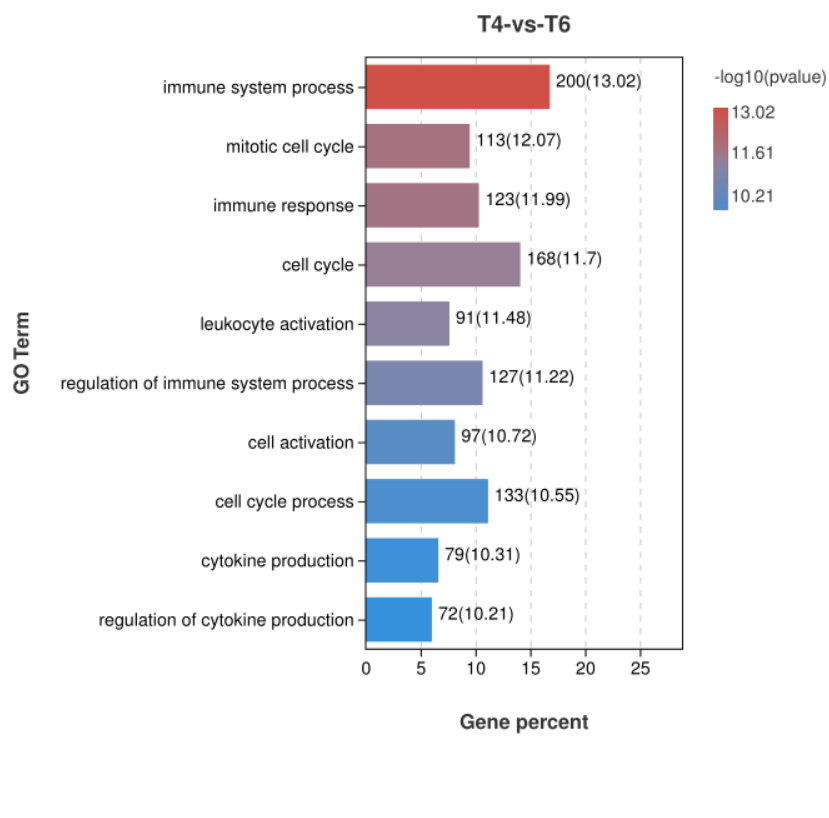


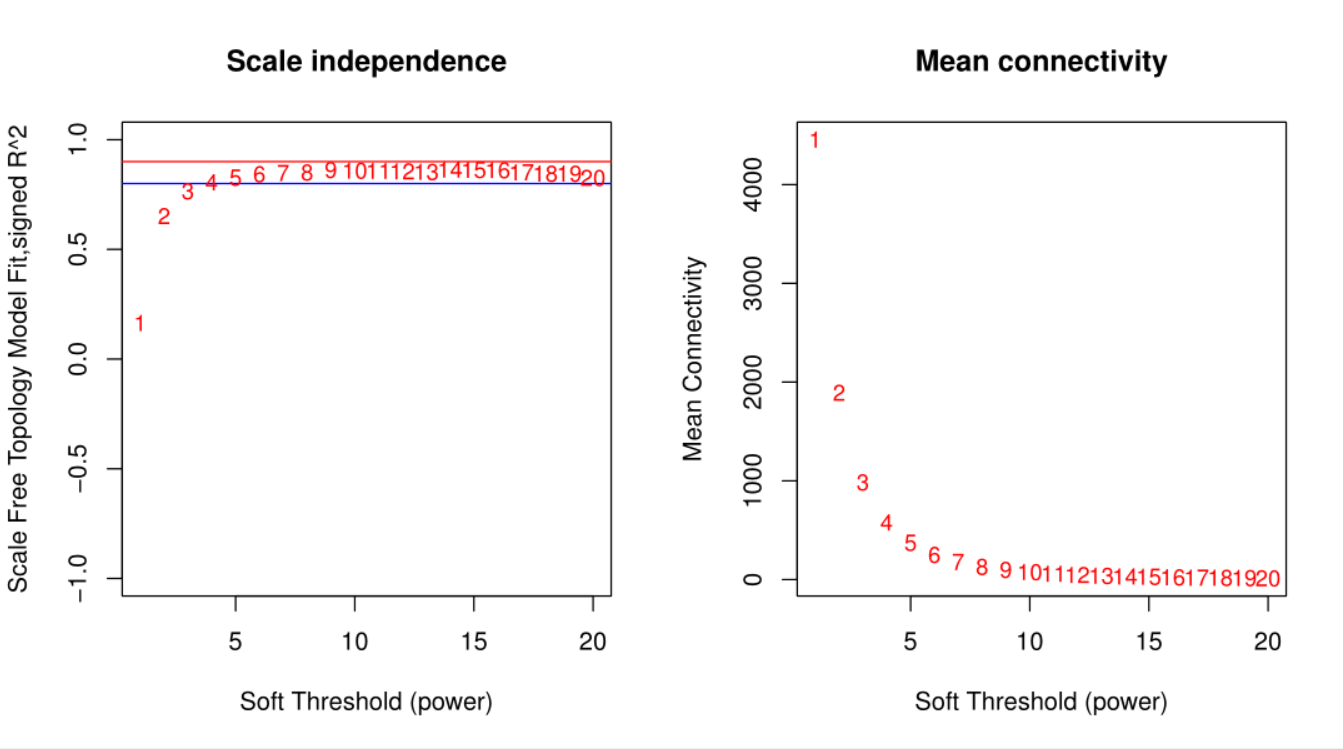
**Supplementary Figure S5** The Power value curve

**Supplementary Figure S6** QC samples are mixed by taking 20 µL from each sample to be tested, which is used to correct the deviation of mixed sample analysis results and errors caused by the analysis instrument itself.


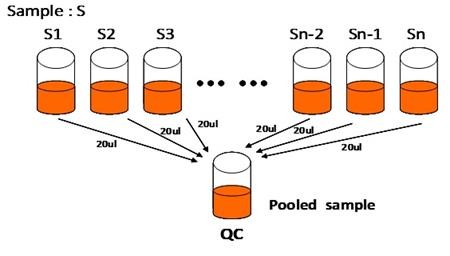


**Supplementary Figure S7** The sorting inspection(POS). The sorting test is a random sorting method used to evaluate the accuracy of OPLS model, which is used to avoid that the classification obtained by supervisory learning method is not accidental


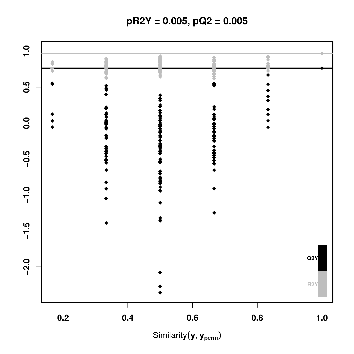

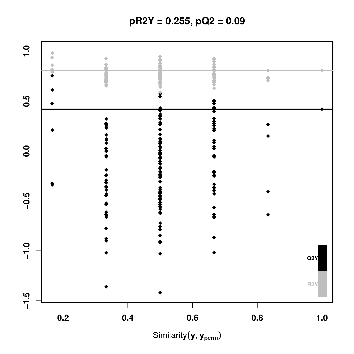

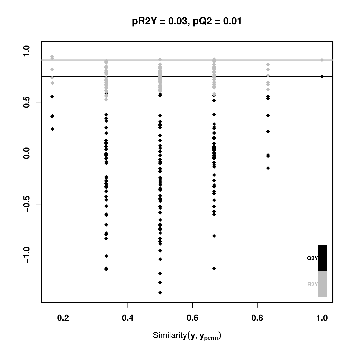

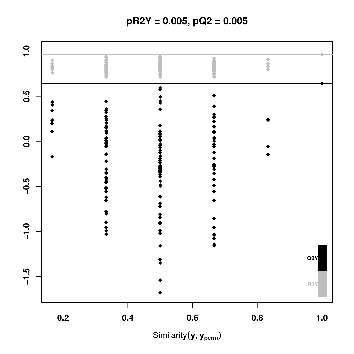


T1-vs-T3

T1-vs-T4

T1-vs-T2

T1-vs-T6

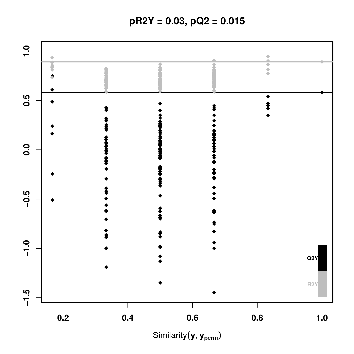

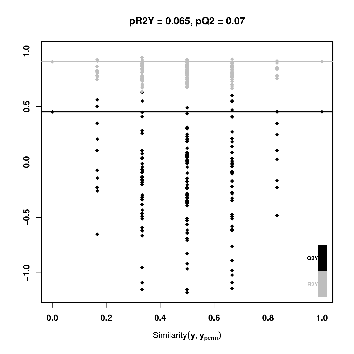

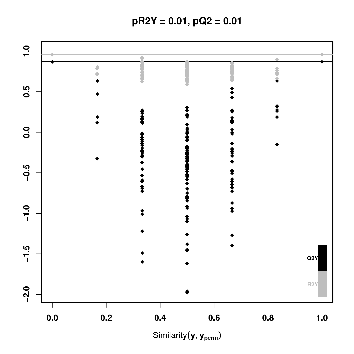

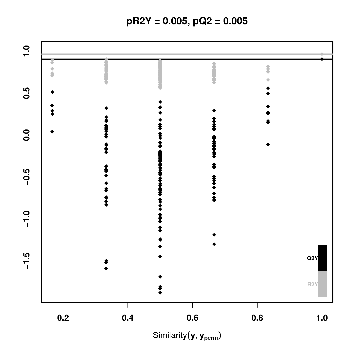


T3-vs-T4

T2-vs-T6

T2-vs-T4

T2-vs-T3

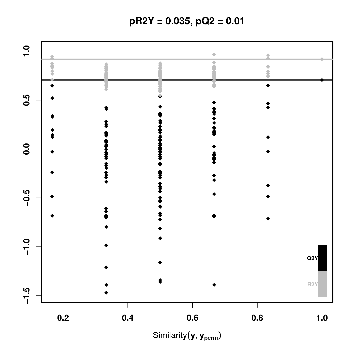

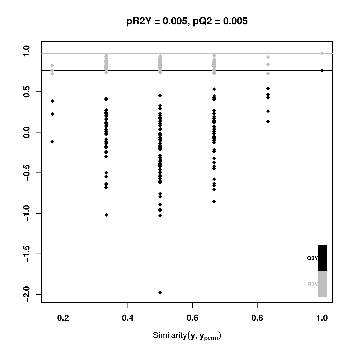


T4-vs-T6

T3-vs-T6

**Supplementary Figure S7** The sorting inspection(NEG)


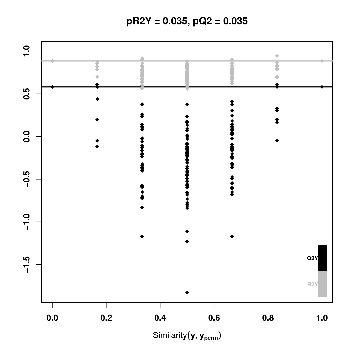

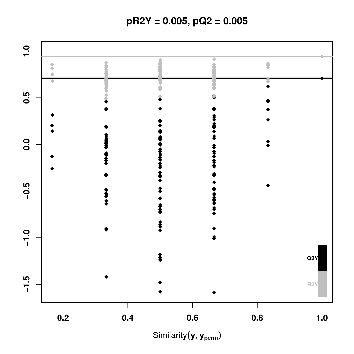

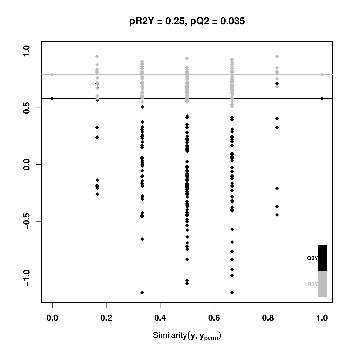

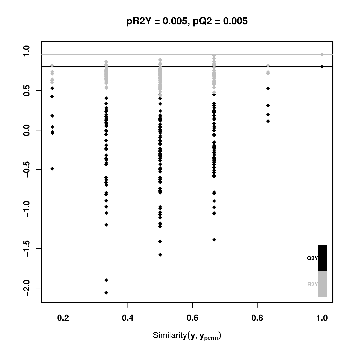


T1-vs-T2


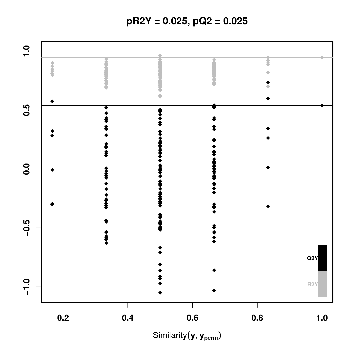

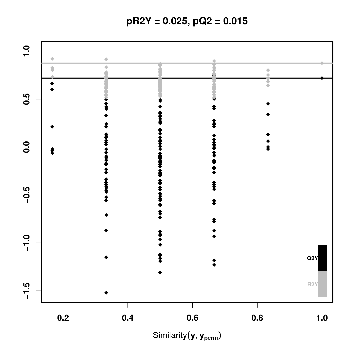

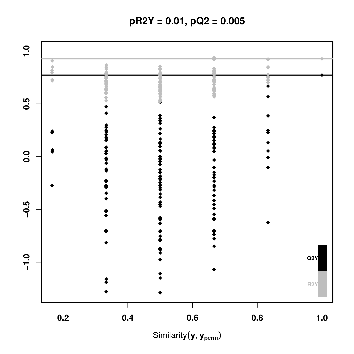

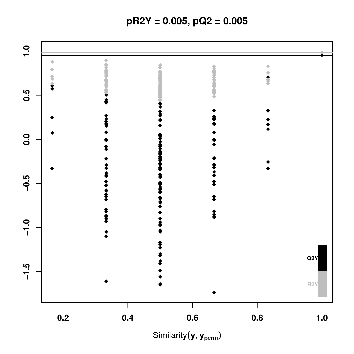

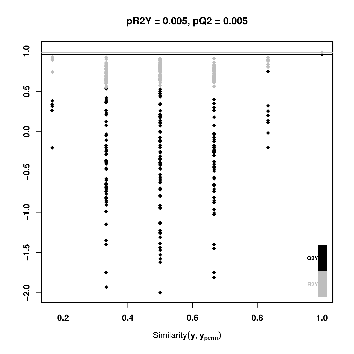

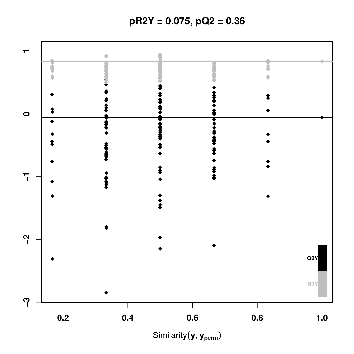


T1-vs-T3

T1-vs-T4

T1-vs-T6

T2-vs-T3

T2-vs-T4

T2-vs-T6

T3-vs-T4

T3-vs-T6

T4-vs-T6
